# Metagenomic Insights Into the Mechanisms for Biodegradation of Polycyclic Aromatic Hydrocarbons in the Oil Supply Chain

**DOI:** 10.3389/fmicb.2020.561506

**Published:** 2020-09-18

**Authors:** Kelly J. Hidalgo, Isabel N. Sierra-Garcia, Bruna M. Dellagnezze, Valéria Maia de Oliveira

**Affiliations:** ^1^Microbial Resources Division, Research Center for Chemistry, Biology and Agriculture (CPQBA), University of Campinas (UNICAMP), Paulínia, Brazil; ^2^Graduate Program in Genetics and Molecular Biology, Institute of Biology, University of Campinas (UNICAMP), Campinas, Brazil; ^3^Biology Department & Centre for Environmental and Marine Studies (CESAM), University of Aveiro, Aveiro, Portugal

**Keywords:** oils reservoirs, sea water, groundwater, bioremediation, PAH

## Abstract

Petroleum is a very complex and diverse organic mixture. Its composition depends on reservoir location and *in situ* conditions and changes once crude oil is spilled into the environment, making the characteristics associated with every spill unique. Polycyclic aromatic hydrocarbons (PAHs) are common components of the crude oil and constitute a group of persistent organic pollutants. Due to their highly hydrophobic, and their low solubility tend to accumulate in soil and sediment. The process by which oil is sourced and made available for use is referred to as the oil supply chain and involves three parts: (1) upstream, (2) midstream and (3) downstream activities. As consequence from oil supply chain activities, crude oils are subjected to biodeterioration, acidification and souring, and oil spills are frequently reported affecting not only the environment, but also the economy and human resources. Different bioremediation techniques based on microbial metabolism, such as natural attenuation, bioaugmentation, biostimulation are promising approaches to minimize the environmental impact of oil spills. The rate and efficiency of this process depend on multiple factors, like pH, oxygen content, temperature, availability and concentration of the pollutants and diversity and structure of the microbial community present in the affected (contaminated) area. Emerging approaches, such as (meta-)taxonomics and (meta-)genomics bring new insights into the molecular mechanisms of PAH microbial degradation at both single species and community levels in oil reservoirs and groundwater/seawater spills. We have scrutinized the microbiological aspects of biodegradation of PAHs naturally occurring in oil upstream activities (exploration and production), and crude oil and/or by-products spills in midstream (transport and storage) and downstream (refining and distribution) activities. This work addresses PAH biodegradation in different stages of oil supply chain affecting diverse environments (groundwater, seawater, oil reservoir) focusing on genes and pathways as well as key players involved in this process. In depth understanding of the biodegradation process will provide/improve knowledge for optimizing and monitoring bioremediation in oil spills cases and/or to impair the degradation in reservoirs avoiding deterioration of crude oil quality.

## Introduction

**FIGURE 1:**
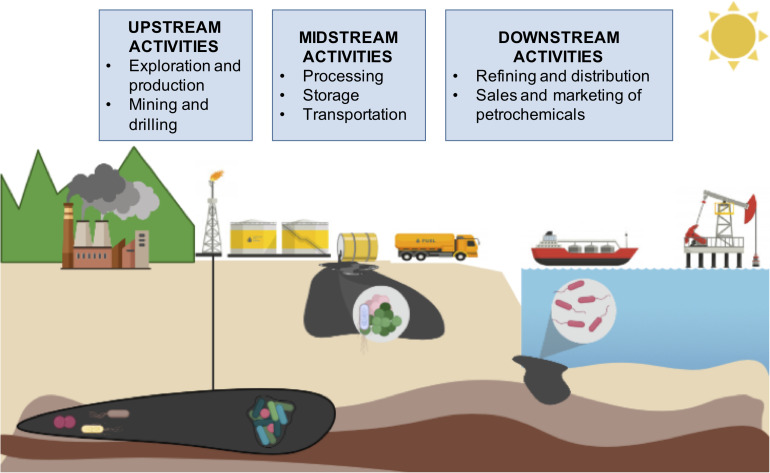
Oil supply chain.

The petroleum industry is one of the largest global industries and covers a vast range of activities across the world. All the activities and processes involved in the petroleum industry are referred to as oil supply chain activities and are divided in three parts (Figure 1): (1) upstream, (2) midstream and (3) downstream. Upstream refers to the origin of the oil: petroleum exploration and extraction. Midstream refers to transportation of raw crude oil through pipelines, rail car and/or tanker to refineries. Downstream describes the refining processes to produce different usable products, like gasoline, diesel, jet fuel and other petrochemicals. One barrel of crude oil (42 gallons) as input can produce 44% of gasoline, 24% of diesel and heating oil, 4% of jet fuel, and 22% of assorted products including petrochemicals, lubricants and asphalt. This manufacturing, refining and petrochemical activities are part of the downstream stage (Big Data and Analytics, 2020). During oil supply chain stages, crude oils are subjected to biodegradation, biodeterioration, acidification and souring. Also, oil or by-products spills are frequently reported affecting not only the environment, but also the economy and human resources.

Petroleum is naturally formed by a process of deposition of algae in marine sediments that for millions of years were subjected to high temperatures and pressures (Hazen et al., 2016). It is a complex mixture of hydrocarbons, many of which are classified as mutagenic, carcinogenic and teratogenic (Liao et al., 2014; Schwarz et al., 2019). Oil hydrocarbons can be classified in four groups according with their solubility in organic solvents and water (Han et al., 2018). These groups are described as: (i) saturated hydrocarbons (or alkanes, or aliphatic), including all the n- and branched alkanes and cycloparaffins, (ii) aromatic hydrocarbons, including monoaromatics such as BTEX (Benzene, Toluene, Ethylbenzene, and Xylenes) and polycyclic aromatic compounds (PAHs), (iii) resins, are compounds that contains sulfur, oxygen and nitrogen and that are dissolved in oil such as quinolines, pyridines, amides and sulfoxides, and (iv) asphaltenes, consisting of aggregates of molecules with naphthenic rings and condensed aromatic connected by paraffin chains (Gentili et al., 2006; Costa et al., 2012; Wu et al., 2013; Abdel-Shafy and Mansour, 2016; Varjani, 2017; Shahsavari et al., 2019). PAHs are one of the major components of crude oil and their by-products are released into the environment during incomplete combustion or by accidental spills over the oil supply chain (Johansson and Van Bavel, 2003). PAHs are organic compounds compose by at least two fused benzene or aromatic rings in linear, angular, or cluster arrangements, resulting in diverse structural configurations (Bamforth and Singleton, 2005; Sharma, 2014; Ghosal et al., 2016). There are two kinds of PAHs, low-molecular-weight (LMW) PAHs, that contain up to two or three rings (naphthalene, acenaphthene, acenaphthylene, fluorene, anthracene, and phenanthrene) and high-molecular-weight (HMW) PAHs, with more than three rings (fluoranthene, pyrene, benzo[a]pyrene, perylene, etc.). HMW-PAHs are more toxic and structurally more stable than the light PAHs (Kuppusamy et al., 2016; Li et al., 2016). The increase in the hydrophobicity, electrochemical stability and resistance toward biodegradation and carcinogenic index, occurs with the increase in the number of rings (Zander, 1983; Harvey, 1998; Mackay and Callcott, 1998; Marston et al., 2001). Due to their complex structure, low water solubility and high hydrophobicity, PAHs tend to be recalcitrant compounds, resulting in their accumulation in the ecosystems and limited availability to biodegradation (Lawal, 2017). In the last decades, due to the increase of the amount of PAHs from natural and anthropogenic resources, there has been an increase of PAHs concentration in the ecosystems (Juhasz and Naidu, 2000). PAHs are largely present as pollutants in diverse ecosystems, such as air, soils, sediments, surfaces and groundwater (Ghosal et al., 2016). The ubiquitous distribution, combined to their toxic, genotoxic, mutagenic and carcinogenic properties, led PAHs to be considered as priority pollutants (Ghosal et al., 2016). The United States Environmental Protection Agency (US EPA) enlisted 16 PAHs as priority environmental pollutants based on their toxicity and abundance (Table 1; U.S. Department of Health and Human Services, 1999; Liu et al., 2001; Zhang et al., 2011; Abdel-Shafy and Mansour, 2016; Lamichhane et al., 2016; Varjani, 2017). Thus, research aimed at removing these PAHs from the environment has gained substantial increase (Ghosal et al., 2016).

**TABLE 1:** Sixteen PAHs priority pollutants by United States EPA.

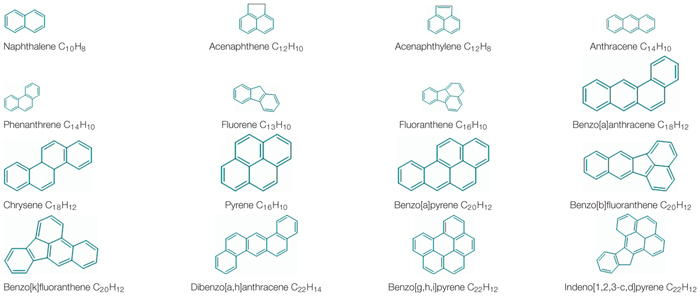

The physicochemical transformations of PAHs in the environment include adsorption, volatilization, photolysis and chemical oxidation. However, microbial degradation is still the most important environmental process which affects the fate of PAHs in contaminated aquatic and terrestrial ecosystems (Lu et al., 2011). Aiming at a remediation approach, PAHs can be transformed by different physical and chemical treatment techniques, like UV oxidation, incineration, solvent extraction and base-catalyzed dechlorination (Gan et al., 2009). However, these techniques have several disadvantages such as cost, complexity, regulation, etc. Additionally, these methods, in many cases, are not efficient enough to completely destroy PAHs molecules, and may instead transform them in intermediates even more toxic (Ghosal et al., 2016). An alternative strategy is bioremediation, that involves the use of the potential of microorganisms to degrade organic pollutants to inoffensive molecules, such a carbon dioxide and water (Zhao et al., 2011; Varjani and Upasani, 2012). This approach appears as an eco-friendly alternative to solve some of the disadvantages of traditional methods. However, the effectiveness of bioremediation processes depends on several factors including the type of contaminant, its bioavailability and the microbial capacity of degradation (Adetutu et al., 2012). Different bioremediation techniques based on microbial metabolism, such as natural attenuation, biostimulation and bioaugmentation are promising approaches to minimize the environmental impact of polluted areas. Microbial hydrocarbon degradation can be performed via different pathways such as phototrophic, anoxygenic, and aerobic or anaerobic chemotrophs pathway (Varjani, 2017) and are limited by multiple factors, like nutrient availability, pH, oxygen content, temperature, PAHs concentration and chemical properties and the type and abundance of microorganisms present in the affected area. Diverse bacterial and fungal species have the potential to degrade/transform PAHs. Microbial communities, metabolic pathways, genes, enzymes and genetic regulation involved in the PAHs degradation have been the focus in PAHs research over the last few decades and have been explored in a great extent (Ghosal et al., 2016). Emerging approaches, such as (meta-) taxonomics and (meta-)genomics can be used to scrutinize and monitoring the diversity and microbial structure, providing access to the taxonomic and functional genes (Thomas et al., 2012). Several studies on hydrocarbon-polluted environments have efficiently used high throughput sequencing techniques to monitor bioremediation processes (Dos Santos et al., 2011; Simon and Daniel, 2011; Coulon et al., 2012; Yergeau et al., 2012a,b; Ma Q. et al., 2015; Wang et al., 2016). Thus, with this modern approaches of molecular biology, the current knowledge about non-cultured microorganisms have been increased (Urgun-Demirtas et al., 2006) and brought new insights on microbial communities populations, metabolic profiles and specific enzymes involved in PAH biodegradation at both single species and community levels in oil reservoirs and groundwater/seawater spills.

The present review provides a global perspective of the current knowledge on PAH biodegradation taking place in diverse environments (groundwater, seawater, oil reservoir) at different stages of oil supply chain, not necessarily in linear order, with emphasis on genes and pathways as well as key players involved in this process.

## Microbial Biodegradation of Polycyclic Aromatic Hydrocarbons

Microorganisms are able to aerobically and/or anaerobically degrade many hydrocarbons (Head et al., 2006; Meckenstock et al., 2016). There are 175 prokaryotic genera belonging to different phyla of Bacteria and Archaea described, and also almost equal number of fungal genera, able to use hydrocarbons as their carbon source (Hazen et al., 2016). Microorganisms able to degrade PAHs are widely distributed in many environments, even in pristine areas, representing up to 0.1% of the microbiota (Margesin et al., 2003; Ghazali et al., 2004; Brooijmans et al., 2009; Uad et al., 2010; Souza et al., 2014; Shen et al., 2015; Varjani et al., 2015; Lamichhane et al., 2016). Nevertheless, in oil-contaminated ecosystems hydrocarbon degraders can dominate the microbial community (Leahy and Colwell, 1990; Greenwood et al., 2009; Varjani and Upasani, 2016). The most affected (polluted) sites are surrounding areas (water and/or soils) of oil refineries, gas plants, air bases, chemical manufacturing facilities and petrol stations (Juhasz et al., 2005; Seo et al., 2009). The development and improvement of the omics approaches have brought a broader understanding of the diversity, distribution and dynamics of microorganisms, as well as of their specific genes and proteins, involved in hydrocarbon degradation in polluted ecosystems (Pieper and Reineke, 2000; Varjani and Upasani, 2013; Ron and Rosenberg, 2014; Varjani, 2014). In the literature there are many reviews about the degradation pathways of several compounds like phenol, BTEX (monoaromatics) and naphthalene, pyrene, phenanthrene and anthracene (PAHs) at the enzyme level (Heider, 2007; Fuchs et al., 2011; Boll et al., 2014; Waigi et al., 2015; Meckenstock et al., 2016; Wilkes et al., 2016).

**FIGURE 2:**
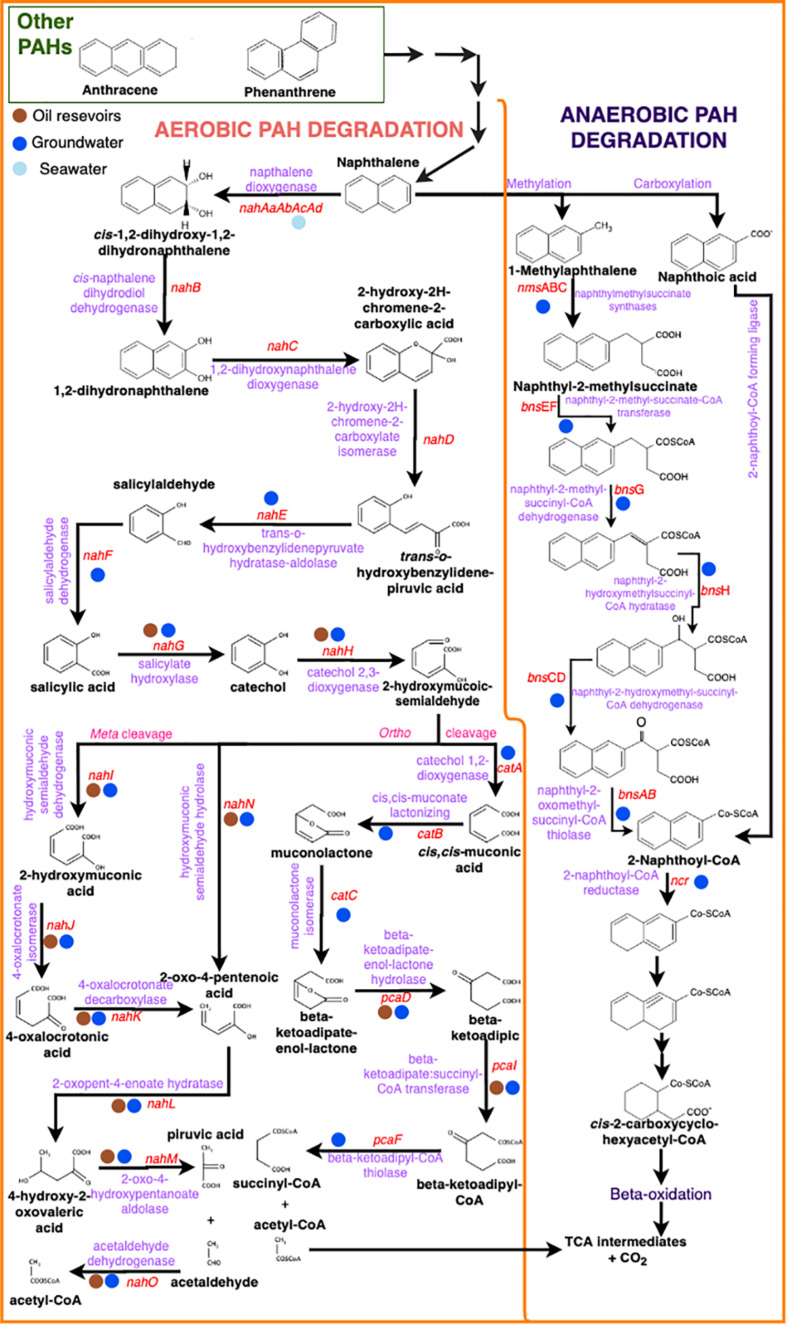
PAH aerobic (left) and anaerobic (right) microbial degradation pathways with intermediate molecular structures (black), enzyme names (purple) and gene names (red). Colored circles represent the presence of the genes in the different environments (brown – oil reservoirs / blue – groundwater / light blue – seawater).

PAH degradation can occur by oxygen-dependent or independent pathways (Figure 2). The aerobic degradation is mainly *via* oxygenase enzymes. The first reaction is an hydroxylation usually catalyzed by a multicomponent dioxygenase enzyme (or ring-hydroxylation dioxygenase-RHD) yielding *cis-*dihydrodiol (Albaigés et al., 1983; Cerniglia, 1993; Saito et al., 1999; Juhasz and Naidu, 2000; Bongiorni et al., 2005; Ghosal et al., 2016). Then, the aromatic ring is rearomatized by the enzyme *cis*-dihydriol dehydrogenase forming dihydroxylated intermediates, which is oxidized to form catechol, the main intermediate of aerobic aromatic hydrocarbon degradation (Shahsavari et al., 2019). The next step depends on the position of the hydroxyl (OH) group in the dihydroxylated intermediates. If the intermediate has the OH group in the *ortho*-position, so an intradiol cleaving dioxygenase will act between the two OH groups yielding *cis*-muconic acid (Juhasz and Naidu, 2000). If the intermediate is in the *meta*-position, cleavage occurs by an extradiol cleaving dioxygenase forming 2-hydroxymuconic semi-aldehyde (Cerniglia, 1993; Shahsavari et al., 2015; Ghosal et al., 2016). Once this process occurs in the first aromatic ring, the second ring is transformed in the same way and so on (Atlas, 1998). At last, transformation of all rings in the PAH molecule results in the production of tricarboxylic acid cycle (TCA) intermediates that can enter in the bacterial central metabolism for further use in the synthesis of cellular constituents and energy (Habe and Omori, 2003). The final products are carbon dioxide and water. The HMW-PAH dioxygenases are encoded by *nid* and *pdo* genes in gram positive bacteria (Gupta et al., 2015). However, several studies demonstrated that PAH-ring hydroxylating dioxygenase genes *pah-rdh*? were more effective than *nidA* gene for detecting and quantifying pyrene-degrading bacteria (Bacosa and Inoue, 2015). The LMW-PAH dioxygenases are encoded by *nah* (naphthalene dioxygenase) genes (Gupta et al., 2015) (Figure 2). These genes have been well described and studied in the gram negative *Pseudomonas* (Habe and Omori, 2003; Zhou et al., 2006). The metabolism of LMW-PAHs degradation is carried out in two main stages: a) upper and b) lower pathways (Figure 2). In the upper pathway, the enzymes are encoded by the *nah*AaAbAcAdBCDEF genes and in the lower pathway by the *nah*GTHINLOMKJ genes (Habe and Omori, 2003; Zhou et al., 2006; Gupta et al., 2015). Also, *pah* and *phn* genes have been found in other gram-negative bacteria, such as in the naphthalene and phenanthrene degrader *Comamonas testosteroni* and in the naphthalene, phenanthrene and anthracene degrader *Burkholderia* sp., respectively (Gupta et al., 2015).

In the anaerobic degradation, compounds other than oxygen act as final electron acceptors, such as nitrate and/or sulfate. The anaerobic degradation of PAHs differs substantially from monoaromatic degradation and has so far been studied only for the diaromatics like naphthalene as a model, mainly due to the fact that this compound is intermediate in the degradation pathway of several PAHs with more than two rings (Von Netzer et al., 2016; Figure 2). PAH anaerobic degradation pathway begins with an initial activation attack. For naphthalene, two main activation mechanisms have been proposed: (i) carboxylation to naphthoic acid (Zhang and Young, 1997), and (ii) methylation to 1-methylnaphthalene followed by fumarate addition to naphthyl-2-methylsuccinate, catalyzed by the naphthylmethylsuccinate synthase (NMS) (Meckenstock et al., 2000; Safinowski and Meckenstock, 2006; Musat et al., 2009). Although some authors have also suggested the hydroxylation as an activation mechanism due to the presence of naphthol in a sulfate-reducing naphthalene-degrading culture (Bedessem et al., 1997), this mechanism has not yet been demonstrated in the anaerobic degradation of naphthalene. The subsequent degradation pathway involves different aryl-CoA reductases (Eberlein et al., 2013a,b). The first two activation mechanisms converge in the central intermediate 2-naphthoyl-CoA and thereafter the aromatic ring is reduced by beta-oxidation-like reaction (Zhang et al., 2000; Annweiler et al., 2002; Phelps et al., 2002). The non-activated ring is dearomatized by the 2-naphthoyl-CoA reductase (NCR), which is a flavoprotein with a flavin mononucleotide cofactor (Von Netzer et al., 2016). Analogous to naphthalene, the activation mechanisms of phenanthrene may be carboxylation, or methylation followed by fumarate addition and oxidation to phenanthroic acid (Von Netzer et al., 2016). Some studies have reported both phenanthrene and naphthalene degradation (Coates et al., 1997; Zhang and Young, 1997; Rockne and Strand, 2001).

As hydrocarbon degradation has been demonstrated under aerobic and anaerobic conditions resulting in mitigation of contamination (Wiedemeier et al., 1999; Yu et al., 2005; Vasconcellos et al., 2010; Shin et al., 2019; Bianco et al., 2020), it is mandatory to improve our knowledge on microbial diversity in hydrocarbon-polluted environments, since the microbial composition and metabolic potential is one of limiting factors of the degradation fate of pollutants in contaminated sites (Lovley, 2003). Currently, the genetic basis of the catabolic pathways of the various PAHs remains poorly understood. Thus, the use of recent approaches as meta-taxonomics and metagenomics offers great potential to contribute with information to elucidate PAH degradation pathways (Brennerova et al., 2009; Nyyssönen et al., 2009; Abbai and Pillay, 2013; Uhlik et al., 2013; Mason et al., 2014; Xu et al., 2014; El Amrani et al., 2015; Loviso et al., 2015; Ma B. et al., 2015; Techtmann and Hazen, 2016; Zafra et al., 2016; Duarte et al., 2017; Muangchinda et al., 2018; Tiralerdpanich et al., 2018; Wilhelm et al., 2018; Xu et al., 2018; Hidalgo et al., 2019). From now on, we will present the information available from several studies carried out to explore PAH biodegradation in natural or impacted environments rich in hydrocarbons. We will focus on microbial community approaches when available or in isolated microorganisms from hydrocarbon impacted environments. Most of these studies focus on naphthalene degradation since, as previously stated, it is a centralized intermediate in the degradation pathway of several PAHs with more than two rings.

## Metagenomics and Catabolic Genes

“Omics” approach has been used to deepen knowledge about the diversity and distribution of PAH degraders as well as their genes and metabolic pathways related to hydrocarbon degradation in several marine environments, such as sediments of the ocean floor, water surfaces, beaches, deep sub-surfaces, as well as in terrestrial environments, as groundwater and soils.

Marine microbial metagenomics can provide an increase of data in marine ecology and oceanography, and several works based on different approaches (metagenomic fosmid/cosmid libraries, Sanger and Next generation sequencing shotgun metagenomics) have been described (Gilbert and Dupont, 2011; Nikolaivits et al., 2017).

The explosion of the Deepwater Horizon (DWH) well from British Petroleum (BP) company, in 2010 in Mississippi, United States, was considered the largest oil spill in history, with nearly five million barrels spilled and spread along 1100 km of the American coast (Atlas and Hazen, 2011). Over time, several studies were performed in order to understand the biological effects of the oil release and its microbial community impact. In one of these studies, Hazen et al. (2010) evaluated the microbial diversity from several oil plume samples (water column) formed in the surroundings of the well and in uncontaminated seawater, employing different molecular, chemical and physiological approaches. The authors observed that most OTUs in oil contaminated seawater were associated to the order *Oceanospirillales*, belonging to Y-Proteobacteria, largely composed of known psychrophilic hydrocarbon degraders including *Oleispira antarctica*, *Oleiphilus messinensi*, and *Thalassolituus oleivorans.* Moreover, they found hydrocarbon degradation genes significantly increased in oil plume samples, such as *phd*CI gene encoding carboxylate isomerase for naphthalene degradation (Hazen et al., 2010). Later, a complementary work focused on the functional role of *Oceanospirillales* and other bacteria, using shotgun metagenomics and metatranscriptomics. The authors could corroborate not only the massive abundance of this group in oil contaminated samples, but also their role as active members in the plume. However, genes involved in degradation of aromatic compounds showed low abundance and low levels of expression when compare to those involved in alkane degradation. This could be explained by the fact that recalcitrant compounds were not actively degraded at the time sampling was performed (Mason et al., 2012). Other works involving the DWH accident based on metagenomics were later carried out also focusing on contaminated sediments, corroborating the microbial shift toward the high abundance of hydrocarbon degraders as Alpha- and Gammaproteobacteria, associated to members of taxa known to degrade hydrocarbons, such as *Rhodobacteraceae, Alteromondaceae*, and *Pseudomonadaceae*, and genes and pathways related to PAH, n-alkane and toluene degradation (Lamendella et al., 2014; Mason et al., 2014). Also, high levels of PAH compounds (above 24,000 mg/kg) were discovered in deep sediments in the area near the wellhead. Metagenomic analysis and functional gene assays of these deep-sea sediments closer to the well (3 km) showed high abundance of deltaproteobacteria and genes related to the anaerobic degradation of aromatic and aliphatic hydrocarbons, like *ass*A and *bss*A, both encoding subunits of glycyl radical enzymes: ASS, linked to alkane degradation (addition of fumarate to alkane), and BSS, related to the addition of fumarate to aromatic hydrocarbons to yield benzylsuccinic acids and benzylsuccinate derivatives, respectively. The detection of benzylsuccinate metabolites provided indication for anaerobic biodegradation of alkylbenzenes *in situ* (Kimes et al., 2014).

Basins in tropical seas have also been studied, such as Campos Basin, an area susceptible to oil contamination in South Atlantic Ocean (Appolinario et al., 2019). The authors investigated the biodegradation potential of marine microorganisms in three different depths: (i) surface (5 m); (ii) intermediate (chlorophyll maximum layer, 80 m); and (iii) near the bottom (1,200 m), using seawater samples supplemented with crude oil and incubated during 52 days. Metagenomics was used to characterize taxonomic and functional microbial diversity and infer microbial abundance related to oil degradation. The genera *Alteromonas* (∼10%) and *Marinobacter* (∼13%) were more abundant in surface and chlorophyll maximum (80 m) samples, respectively, whereas *Colwellia* (∼24%) was the most abundant in bottom samples. The genus *Colwellia* has been reported as alkane and aromatic degrader (Mason et al., 2014; Campeão et al., 2019) and results found by Appolinario et al. (2019) could suggest a higher oil-degrading potential of such bacteria in deep seawater. Moreover, genes associated with metabolism of aromatic compounds, as naphthalene and phenanthrene, were also monitored and reached a peak at the end of 52 days of experiment (Appolinario et al., 2019). In a previous work from our group, a consortium consisting of metagenomic clones and a *Bacillus* strain, both recovered from petroleum reservoir, were employed in bioaugmentation experiments using artificially petroleum contaminated seawater from Messina harbor (Italy) in mesocosms scale (3000 L). After 30 days, aromatic degradation was more effective in the bioaugmentation treatment compared to control, with biodegradation rates ranging from 70 to 99%. Autochthonous community dynamics varied between treatments throughout the experimental days, and although the microorganisms added to the bioaugmentation treatment were not detected in high abundances, the consortium was shown to contribute to a significant increase in aromatic hydrocarbon degradation (Dellagnezze et al., 2016). Metagenomics and cultivation-based studies from polluted environments reporting genes associated to PAH degradation have been extensively described in literature (Table 2).

**TABLE 2:** Genes related to PAH degradation in oil reservoirs, groundwater and marine environments.

**Gene**	**Microbial origin**	**Source**	**References**
*nah*AaAbAcAd cluster, *nah*EFGHIJKLMNO *cat*AB *pca*DFI	Metagenome	Jet-fuel contaminated aquifer (Brazil)	Hidalgo et al., 2019
*nms*ABC *bns*ABCDEFGH ncr	Metagenome	Creosote-polluted groundwater	Nyyssönen et al., 2009; Meckenstock et al., 2016
*bss* (benzylsuccinate synthase)	Metagenome	Deep sea sediments (Gulf of Mexico)	Kimes et al., 2013
*bss* (benzylsuccinate synthase)	Metagenome Assembled Genomes (MAGs)	Alaska North Slope oil fields	Hu et al., 2016
*bss* (benzylsuccinate synthase)	Metagenome Assembled Genomes (MAGs)	Petroleum reservoirs Brazil	Sierra-Garcia et al., 2020
*pah*A1-4 gene cluster (dioxygenase)	*Cycloclasticus s*p. 78-ME	Mediterranean Sea (Italy)	Messina et al., 2016
*nah*Ac/NDO (naphtalene dioxygenase)	*Alteromonas* sp. strain SN2	Contaminated sea (South Korea)	Jin et al., 2012
*cat*E (catechol-2,3-dioxygenase)	*Limnobacter* sp. Metagenome Metagenome	Baltic Sea (Estonia/Finland) Deep sea (Australia) Bohai Sea, China	Vedler et al., 2013; He et al., 2016; Van De Kamp et al., 2019
*phn*Ac (phenanthrene dioxygenase)	Metagenome	Ushuaya Bay (Argentina) Zhoushan Archipelago, China	Marcos et al., 2009; Peng et al., 2020
*car*ABC (carbazole 1,9a- dioxygenase)	*Neptuniibacter* sp. strain CAR-SF	Isolate from seawater Japan	Nagashima et al., 2010
LmPH (multicomponent phenol hydroxylase)	*Limnobacter* sp.	Baltic Sea (Estonia/Finland)	Vedler et al., 2013

Oil supply chain associated activities are extensive to some polar regions, making them susceptible to petroleum hydrocarbon contamination. Thus, studies focusing on these environments are highly relevant in order to improve/optimize bioremediation strategies. Nonetheless, the management in these regions encounters some challenges due to intrinsic harsh conditions. In 2008, a survey was carried out aiming at bioprospecting marker genes of PAH degradation by using Aromatic Ring-Hydroxylating Dioxygenase (ARHD) gene libraries obtained from sediments of the Patagonia Coast, Argentina. The authors could find eight different ARHD gene types, with five of them showing no close relatives in the databases. The remaining three were associated to *nah*A*c*-like and *phn*Ac-like genes, related to naphthalene and phenanthrene degradation, respectively, as described in *Alcaligenes faecalis* AFK2, and to *phn*A1-like genes from marine bacteria belonging to *Cycloclasticus* genus (Lozada et al., 2008). Later, the same authors investigated functional targets for PAH degradation in chronically polluted subantarctic marine sediments, in Ushuaya Bay, Argentina. Based on the use of primers designed for the gram negative bacterial dioxygenase genes, the authors identified 14 different groups of genes, most of them significantly related to dioxygenases from gram positive bacteria belonging to genera *Bacillus, Rhodococcus, Mycobacterium, Nocardioides*, and *Terrabacter* (Marcos et al., 2009).

In the Arctic Ocean, a study evaluated the presence of PAHs and their bioattenuation in the open sea. Samples from 19 sediment cores (deep sea sediments) were collected from Canada Basin, the Chukchi Plateau, Alpha Ridge and Makarov Basin and evaluated by 16S rRNA gene large scale sequencing to determine the diversity of bacteria involved in PAH degradation *in situ*. The potential degrading groups observed were members of the genera *Pseudomonas, Pseudoalteromonas, Cycloclasticus, Halomonas, Bacillus, Colwellia, Marinomomas, Salinisphaera, Shewanella, Alcanivorax, Dietzia*, and *Acinetobacter*, being the genus *Dietzia* widespread in all sediment samples and the most abundant group (Dong et al., 2015). In another study, Mcfarlin et al. (2014) evaluated the biodegradation of crude oil carried out at −1°C using seawater mesocosm from Chukchi Sea, Alaska, simulating natural water column. Surfactant Corexit 9500 was added along with oil in order to biostimulate degradation. By the end of experiment, the indigenous microbial community was capable to biodegrade chrysene, a four-ringed PAH. Nevertheless, the degradation rates were higher for the lower molecular weight compounds, such as naphthalene and phenanthrene, demonstrating that the indigenous microbiota from Arctic seawater is capable of performing extensive biodegradation at low environmental temperature (Mcfarlin et al., 2014).

## PAH Biodegradation in Upstream Operations: Petroleum Reservoirs

Both traditional microbiological methods and *omics* approaches have been widely used to assess microbial community degraders and hydrocarbon biodegradation potential in petroleum and gas industry-associated environments. These scientific efforts have started long time ago aiming at a deeper understanding of the role of microorganisms in petroleum deterioration, as well as at bioprospecting specific properties of indigenous microorganisms for improving/optimizing biotechnological processes, such as Microbial Enhanced Oil Recovery (MEOR) (Röling et al., 2003; Youssef et al., 2009; Wentzel et al., 2013; Pannekens et al., 2019).

In oil industry, upstream operations include exploring, drilling and bringing to the surface oil resources from petroleum reservoirs. Equipment in upstream oil industry operations such as pipelines, vessels and others, integrate a broad environment were microorganisms predominantly anaerobes thrive. The existence of microorganisms in petroleum reservoirs and associated facilities is well known for those working in oil industry and petroleum microbiologist. In fact, the study of microbiology in petroleum systems worldwide has been encouraged by the operational and economic consequences of microbial activities in the petroleum systems.

Biodegradation of petroleum hydrocarbons is one of the microbial activities in oil reservoirs that has major implications in the properties and quality of oil and, consequently, its production and value. In the *in reservoir* microbial degradation process, lighter fractions of petroleum hydrocarbons such as saturated hydrocarbons and light aromatic hydrocarbons are consumed, leading to an increase of the proportion of branched and cyclic hydrocarbons, heavier aromatic hydrocarbons and polar fractions of oils such as resins and asphaltenes (Head, 2017). There is also an increase in the concentration of recalcitrant compounds, commonly referred to as “unresolved complex mixture” (UCM). The resulting heavy oils have physical and chemical properties that make them more difficult and costly to be produced and refined (Head et al., 2010; Head, 2017).

The microbial degradation of lighter hydrocarbons in worldwide petroleum reservoirs has led to the transformation of conventional oils to heavy -unconventional- oils which are characterized by high viscosity and low API gravity. Heavy oils are distinguished from light oils mainly by the API gravity values. Definition of heavy oils is often applied inconsistently to crude oil, sometimes it is applied to API gravity of less than 20, but other definitions embrace gravities less than 22 or less than 25 (Speight, 2015). In general, the exploration and extraction of petroleum resources is preferably conducted from better quality and accessible resources like conventional oils, before progressing to lower quality, lower API and less accessible resources that require more efforts and higher economic and environmental costs (unconventional oil resources) (Nduagu and Gates, 2015). Nevertheless, heavy oils dominate the reserves of petroleum around the world, estimated to approach 5.6 trillion barrels (bbl), predominantly located in the western hemisphere (Hein et al., 2013; Head et al., 2014).

Petroleum reservoir microbiology and reservoir fluid chemistry indicate that oil reservoirs are primarily anoxic environments (Head, 2017). Therefore, the current understanding is that biodegradation of oil and formation of heavy oils is the result of anaerobic metabolism of microorganisms living in the subsurface environments (Wentzel et al., 2013; Li et al., 2017; Sierra-Garcia et al., 2017). However, current knowledge of the pathways for anaerobic hydrocarbon degradation in petroleum reservoirs is scarce. In fact, aerobic heterotrophs (or in some cases facultative) organisms are continuously reported in literature, such as *Bacillus* spp., *Acinetobacter* spp., and *Pseudomonas* spp., being detected or isolated from subsurface hydrocarbon-rich reservoir environments (Orphan et al., 2000; Da Cruz et al., 2011; Berdugo-Clavijo et al., 2012; Li et al., 2012; An et al., 2013; Meslé et al., 2013; Gieg et al., 2014). Previous studies from Da Cruz et al. (2011) have proposed that oil degradation could be a combined accomplishment of both aerobic and anaerobic bacteria living in consortia. Similarly, a metagenomic approach applied by An et al. (2013) revealed that aerobic hydrocarbon-degrading related genes and bacteria are highly abundant in a wide range of hydrocarbon-rich environments (coal beds, tailing ponds, waters from oil reservoirs, etc.). In addition, a functional-based metagenomic study from reservoir samples showed that hydrocarbon degradation activities expressed by metagenomic fosmid clones were coded by fragmented gene clusters from aerobic and anaerobic degradation pathways occurring in the same fosmid inserts (Sierra-García et al., 2014).

Specific literature on the *in reservoir-*degradation of larger and recalcitrant compounds such as PAH and its influence on oil viscosity is limited (Xia et al., 2016). Conversely, for saturated hydrocarbons such as alkanes, biodegradation is now widely accepted to occur anaerobically by syntrophic n-alkane degradation and methanogenesis. In many studies, the ability of anaerobic enrichment cultures derived from oil fields to degrade saturated hydrocarbons has been reported (Zengler et al., 1999; Siddique et al., 2006; Jones et al., 2008; Gieg et al., 2010; Mbadinga et al., 2011; Wang et al., 2011; Zhou et al., 2012; Tan et al., 2013). This is not the case for PAH degradation, which is scarcely reported in samples originating from oil reservoirs. An early study observed that microbial community structure changes during the progressive degradation of oil and the removal of n-alkanes (Hallmann et al., 2008). In this case, parallel to the removal of n-alkanes, bacterial biomass increases, and diversity differs from that of the alkane degrading community. Therefore, degradation of recalcitrant compounds in oil reservoirs is thought to involve hydrolytic and fermentative bacteria that carry a wider range of metabolic capabilities (Röling et al., 2003; Hallmann et al., 2008).

Considering that biodegraded oils have higher content of cyclic and heavier aromatic hydrocarbons compared to undegraded oils, the study of biodegraded oil reservoirs may offer fundamental insights into the aerobic and/or anaerobic nature of the polycyclic hydrocarbon biodegradation in such environments. Metabolites indicative of anaerobic degradation of naphthalene and polyaromatic hydrocarbons have been detected in biodegraded oils (Aitken et al., 2004) and production fluids from oil reservoirs (Bian et al., 2015). Particular microbial community analyses in highly biodegraded oil fields, have shown the dominance of specific bacterial groups like Epsilonproteobacteria (Voordouw et al., 1996; Grabowski et al., 2005; Hubert et al., 2012). Some previous comparative studies between degraded and non-degraded oils apparently did not show significant differences in microbial communities between those reservoirs using ARDRA analysis (Sette et al., 2007). However, more recent research, based on higher resolution techniques, has shown differences between biodegraded and non-degraded petroleum samples where biodegraded oils contain higher microbial diversity (Hallmann et al., 2008; Silva et al., 2013; Sierra-Garcia et al., 2017). This is in accordance with the fact that it is expected that the microbial populations able of degradation of light oils, rich in saturated hydrocarbons like n-alkanes, are different from the ones associated with heavy oils, containing high levels of aromatic hydrocarbons (Head et al., 2014).

Recently, the microbiome associated with biodegraded and non-biodegraded oil reservoirs was analyzed in a more comprehensive and thorough study using whole shotgun metagenomic approach (Sierra-Garcia et al., 2020). The analysis showed that the biodegraded oil encompassed equal abundance of bacteria and archaea together with higher proportion of genes corresponding to anaerobic hydrocarbon degradation. On the other hand, the reservoir containing non-degraded oil was dominated by bacteria mainly associated with *Marinobacter* and less proportion of archaea together with lower frequency of genes of anaerobic hydrocarbon degradation. These results reinforced the important role of syntrophic interactions between bacteria and archaea to perform biodegradation of petroleum components in oil reservoirs through the processes of anaerobic metabolism of hydrocarbons and methanogenesis (Zengler et al., 1999; Gieg et al., 2014; Sierra-Garcia et al., 2020).

Although diversity and physiology of microorganisms inhabiting oil fields have been compiled and reviewed worldwide (Wentzel et al., 2013; Li et al., 2017; Sierra-Garcia et al., 2017), determining if the microorganisms recovered are indigenous to the subsurface environment and not from contamination sources is a thorny issue in petroleum microbiology. The reason is because several of the microbial community studies in petroleum reservoirs have analyzed produced waters and thus, there are some drawbacks to consider when interpreting petroleum microbiology. Firstly, because of the nature of the produced waters and water injection practices, some of the microorganisms detected in such are considered to be contaminants (Head et al., 2014). Secondly, there is an increasing evidence that microbial life can exist in the oil itself and even that the microbial communities associated with the different components of the oil fluids (e.g., crude oil or production or formation waters) could be different (Kryachko et al., 2012; Meckenstock et al., 2014; Wang L.-Y. et al., 2014; Cai et al., 2015). From these studies, it seems that bacterial communities and functional genes are more abundant and diverse in the oil phase than in the water phase (Kobayashi et al., 2012; Wang L.-Y. et al., 2014; Cai et al., 2015; Sierra-Garcia et al., 2020).

Regardless of the origin of the microbial life in petroleum reservoirs, the *in-situ* activities of indigenous microorganisms have significant consequences for petroleum systems. Before drilling or during oil production, microorganisms are interacting and responding to the availability of carbon sources, nutrients, electron donors and other biotic and abiotic conditions (Head, 2017). For example, when sulfate is abundant, sulfate reduction is a significant driver of the petroleum biosphere. But the abundance of sulfate reducing bacteria (SRB) is considered an artifact of seawater injection in petroleum reservoirs. SRB mostly depend on small organic substrates such as volatile fatty acids and hydrogen, as electron donors (Hallmann et al., 2008). In petroleum reservoirs where no seawater injection for secondary recovery has been practiced, the microbiome is mainly associated with fermentation reactions carried out by syntrophic bacteria that deliver hydrogen, carbon dioxide and acetate to methanogens (Head, 2017).

Sierra-Garcia et al. (2020) compared the microbiome of crude oils in two production wells, one that been had been exposed to water flooding for secondary recovery and the other one considered pristine (no water flooded). In this study, evidences for the ongoing oil biodegradation of the pristine reservoir were found in the taxonomic and functional composition of the reservoir microbial community, where fermentative bacteria of the genera *Syntrophus* and *Syntrophomonas* and members of the phyla Synergistia and Candidatus Atribacteria carried the genes for hydrocarbon degradation in close association with methanogenic bacteria. At the same time, the microbiome of the water flooded oil was dominated by *Marinobacter*-like organisms with a wider heterotrophic metabolism including genes for oxygen reduction, and for sulfate and nitrogen metabolism. In this reservoir, the anthropogenic perturbation with seawater injection was assumed to alter the indigenous microbial community toward fast-growing opportunists that had little effect on the hydrocarbon degradation due to the likely use of other easily available carbon sources or due to the combination of high salinity and temperature limiting *in situ* conditions (Sierra-Garcia et al., 2020). Generally, water injection practices lower temperatures, increase salinity (in seawater injections), and promote the enrichment with exogenous chemicals/nutrients and/or microorganisms, stimulating changes in indigenous microbial communities (Pannekens et al., 2019). Therefore, besides the well-known factors that limit life (nutrient availability, metabolic products, temperature and salinity), indigenous microbial communities in oil reservoirs are affected by the anthropogenic factor, that for a long time has exploited the deep geological resources leading to changes in microbial ecology and activity, ultimately resulting in the modulation of beneficial and/or detrimental microbial processes (Pannekens et al., 2019).

## PAH Biodegradation in Midstream Activities: Consequences on Marine Environment

Independently of the petroleum extraction method (primary or secondary recovery), and of the oil type (light or heavy), after drilling, the oil is transported through pipes to tankers or to oil terminals and then to refineries. During transport, accidental release of crude oil or its derivatives can occur, including tanker spills, explosions and ruptures of pipelines, which can lead to the spill of large volumes of pollutants on the sea surfaces or sub-surfaces. Such oil contamination events result in PAH acute pollution of the marine environment (Duran and Cravo-Laureau, 2016; Nelson and Grubesic, 2018).

Oil pollution brings several consequences, not only environmental impact with contamination of the whole food chain, from phytoplankton to large mammals, but also economic and social impacts in the affected region (Perelo, 2010; Taleghani and Tyagi, 2017). Depending on the dimension of the oil spill, several human activities may be affected as fisheries and mariculture, tourism and recreational facilities, shipping and salt production (ITOPF, 2011; Court et al., 2017). Some of these accidents and their consequences have been vastly reported, such as Exxon Valddez, Alaska, in 1989 (Atlas and Hazen, 2011), Amoco Cadiz, France in 1978 (Ward et al., 1980) and Deepwater Horizon, United States in 2010 (Kimes et al., 2014; Lamendella et al., 2014).

In marine environments, petroleum derivatives undergo a process called weathering, i.e., a series of biological and physicochemical transformations (Mcgenity et al., 2012). Physicochemical processes include evaporation, dissolution, dispersion, emulsification, photo oxidation, adsorption and sedimentation. Biological weathering occurs through microbial metabolism and intake by other organisms (Hassanshahian and Cappello, 2013). The more complex the structure of the hydrocarbon molecule, the more difficult its degradation, hence, saturated hydrocarbon degrades faster compared to aromatics and asphaltenes (Xue et al., 2015).

The restoration of the impacted area involves the attenuation of toxic compounds to reach bearable level and lays on several biotic and abiotic factors, including availability of colonizing microorganisms, biological and climate, among others (Kingston, 2002; Chen and Denison, 2011).

In order to identify possible microbial degraders and unravel their metabolic potential, studies on the characterization of marine bacterial communities have been reported (Atlas and Hazen, 2011; Neethu et al., 2019). These studies have greatly contributed to the identification of key organisms capable to degrade contaminants mainly for applying new *in situ* bioremediation approaches (Hassanshahian and Cappello, 2013).

The release of oil and its derivatives into seawater can cause changes in autochthonous marine microbial communities leading to successive blooms of some bacterial groups observed at low or undetectable levels before the polluting event. These groups composed by particularly specialized obligate hydrocarbon utilizers are defined as “obligate hydrocarbonoclastic bacteria (OHCB)” or “specialized hydrocarbonoclastic bacteria (SHCB) (Yakimov et al., 2007; Teramoto et al., 2013). *Alcanivorax* spp., described as alkane degraders; *Cycloclasticus* spp., as degrading PAHs; *Marinobacter* and *Thalassolituus*, also present in cold environments, are some of these specific hydrocarbon degrading genera. When an oil spill event occurs, approximately 90% of the microbial community are compounded by obligate hydrocarbon-degrading bacteria, extremely relevant in the natural attenuation of oil-polluted marine environments (Yakimov et al., 2007; Mcgenity et al., 2012). The OHCB are broadly distributed; however, some of the species, as *Oleispira antarctica*, have only been detected in cold ocean. Moreover, the type of hydrocarbon contamination can influence microbial shifts, selecting specific genera, such as aliphatic-degrading *Alcanivorax* and aromatic- degrading *Cycloclasticus* (Berthe-Corti and Nachtkamp, 2010).

Nevertheless, other bacteria not associated to obligate or specific hydrocarbon degrading groups have been related to hydrocarbon degradation in marine environments, such as *Bacillus* (Zhuang et al., 2002; Hentati et al., 2016), *Pseudomonas, Acinetobacter* (Tarhriz et al., 2019), *Alcanivorax*, *Marinobacter* and *Rhodococcus* (Wang W. et al., 2014; Catania et al., 2015) *Dietza* and *Rhodococcus* (Wang W. et al., 2014; Yang et al., 2017).

As previously described, some parameters are taken into account as modulators of PAH degradation. Temperature is a key factor, especially in polar and temperate regions. Low temperature influence oil weathering processes and metabolic activity of microorganisms, hindering the biodegradation process or reducing it at extremely low rates (Naseri et al., 2014). This can be a great concern for the delineation of bioremediation strategies in cold oil polluted areas as Arctic regions, rich in hydrocarbon resources and exploited since 1970 by offshore drilling activities.

## PAH Biodegradation in Downstream Activities: Groundwater Spills

The risk of petroleum spills during downstream activities (refining and distribution), including leakages of underground tanks, represents a major factor of groundwater contamination. These pollutants can migrate and reach the groundwater causing serious contamination of water resources. In some cases, released hydrocarbons are distributed as light non-aqueous phase liquid (LNAPL). Although hydrocarbons are known as “immiscible”, they have a very low miscibility in concentrations at the order of micrograms up to a few grams per liter. Thus, the LNAPL can continuously release hydrocarbons to the aqueous phase, leading to a rapid dissolved phase plume formation with high potential toxicity (Coates et al., 2002; Chakraborty and Coates, 2004).

Commonly, due to the rapid oxygen depletion by aerobic hydrocarbon degraders, the hydrocarbon-polluted aquifers are anaerobic. As previously described, in the anaerobic degradation, microorganisms use alternative final electron acceptors (e.g., iron, sulfate or nitrate), frequently in a syntrophic relationship with other bacteria and methanogenic archaea (Van Hamme et al., 2003; Keller et al., 2018).

The -omics approaches have been used by several authors for monitoring/improving bioremediation process. The taxonomic and functional microbial diversity have been characterized in aquifer sediments collected in the saturated zone and in *in situ* microcosms enriched with some mono- and di- aromatic hydrocarbons (toluene, benzene and naphthalene) from jet fuel, revealing the key microorganisms involved in the natural attenuation of these compounds. In *in situ* microcosms, b-diversity analyses showed a clear difference between the microbiome of the microcosms enriched with monoaromatics and diaromatics, confirming the hydrocarbon-degrading microorganisms have preference for each class of molecule. Families like SR-FBR-L83 (Ignavibacteriales order), Syntrophaceae and Spirochaetaceae were almost exclusive in the *in situ* microcosm amended with naphthalene. On the other hand, Geobacteraceae and Peptococcaceae were the most relevant families in the toluene and benzene amended microcosms (Hidalgo et al., 2019). Syntrophaceae and Spirochaetaceae have been reported as naphthalene degraders under sulfate conditions (Kümmel et al., 2015). Assessment of the functional profiles revealed that genes related with all stages of monoaromatic anaerobic degradation were found, including genes involved with carboxylation and fumarate addition for the initial activation mechanism. In aquifer sediments, genes of the later stages of naphthalene aerobic degradation pathway (catechol conversion) (Figure 2 and Table 2), mainly *via* dioxygenases (*ortho* cleavage; *nah*F, *nah*G, *nah*I, *nah*J, *nah*K, *nah*N, *nah*L, *nah*M, *nah*O, *cat*A, *cat*B, *cat*C, *pca*D, *pca*I, *pca*F) were found, especially in the border of the contamination plume, where oxygen concentration was higher. These genes were mostly affiliated to *Acinetobacter* genus. However, genes for anaerobic degradation of naphthalene were not found. Based on the results, the authors concluded that the affected area was able to decontaminate monoaromatic hydrocarbons by natural attenuation due to the presence of degrading taxa and genes related with degradation of the contaminant. On the other hand, for naphthalene decontamination, other bioremediation approaches, such as biostimulation with electron acceptors like nitrate and/or sulfate for anaerobic microbiota or oxygen for aerobic microbes, should be necessary (Hidalgo et al., 2019).

Ramos et al. (2014) investigated microbial communities related to aromatic hydrocarbons bioremediation by methanogenic syntrophic biodegradation in groundwater polluted with biodiesel mixture (diesel/biodiesel) through controlled field experiment. They used ammonium acetate to biostimulate the methanogenic metabolism and compared the microbial community shifts using 16S rRNA gene pyrosequencing. They observed an increase in the relative abundance of *Desulfitobacterium* and *Geobacter* spp., which are known as anaerobic hydrocarbon degraders, demonstrating the importance of enriching hydrocarbon degrading microorganisms by a syntrophic process with methanogenic archaea to enhance the biodiesel biodegradation (Ramos et al., 2014). In the same polluted site, Müller et al. (2017) used ammonium acetate and a low-cost sustainable product recovered from acid mine drainage treatment to stimulate iron and sulfate reduction metabolisms. Amplicon 16S rRNA sequencing analyses showed a shift in the community after 3 months of treatment and almost 100% of the population were affiliated to *Geobacter* genus in 7.4 months. This genus has been described as dominant member in microbial communities with high iron concentration (Botton et al., 2007; Lin et al., 2009) and they can degrade organic compounds coupled to iron reduction (Anderson et al., 1998; Botton et al., 2007; Maithreepala and Doong, 2009; Zhang et al., 2012; Li et al., 2014). These results were in accordance with the high iron (II) concentration and the acetate depletion observed. Thirteen months later, almost 60% of the sequences were assigned to GOUTA19 genus (family Thermodesulfovibrionaceae). GOUTA19 genus was also observed before in soil irrigated with water contaminated with acid mine drainage (Sun et al., 2015), oil storage tanks (Watanabe et al., 2002) and in monochlorobenzene-contaminated water (Alfreider et al., 2002). Some members of the Thermodesufovibrionaceae family have been related with sulfate reduction process (Rabus et al., 2006; Bhatnagar et al., 2015; Sun et al., 2015). The lower concentration of benzene and naphthalene compared with the control (without stimulation) over the whole experiment supports the key role of the genera *Geobacter* and GOUTA19 in the degradation of these aromatic hydrocarbons by iron and sulfate reduction metabolisms, respectively (Müller et al., 2017).

The efficacy of the bioaugmentation approach was investigated in a large-scale treatment of petroleum contaminated groundwater in a petroleum facility. The bioaugmentation consortium composed by 22 aerobic bacterial strains was obtained from a biofilter of a wastewater treatment plant located in the petroleum facility. The treatment was performed on a modified aerated ISO tank (18 m^3^ of capacity). The contaminated groundwater was pumped into the ISO tank and 16 L of the microbial consortium was added. Total petroleum hydrocarbons (TPH) concentration decreased from 1,563 mg.L^–1^ to 89 mg.L^–1^. The microbial diversity and composition were assessed by 16S rRNA sequencing, which showed a first shift in the microbial profile when the consortium was inoculated but after 18 days of treatment a stable microbial community was observed. The results suggested that indigenous bacteria growth, overtaken the survival rate of some strains of the consortium (e.g., bacilli). This might have played an important role in the continuous degradation of TPH. At the beginning of the treatment, the genera *Cloacibacterium, Sediminibacterium, Brevundimonas* and *Curvibacter* were the most abundant. After the 18th day, the predominant genus was *Flavobacterium* followed by *Sediminibacteium* and *Limnobacter.* At the end of the treatment *Flavobacterium* was the dominant genus accounting for almost 41% of the community (Poi et al., 2018). This results supply evidence for the use of bioaugmentation as an option for the treatment of large volumes of hydrocarbon-polluted groundwater and corroborates previous findings on the metabolic potential for PAH degradation of the consortia members (Poi et al., 2018). Previously, *Cloacibacterum* spp. have been reported in fresh water microcosms contaminated with naphthalene (Aburto et al., 2009), whereas in aerobic reactors for treatment of PAH-contaminated soil, *Sediminibacterium* members was observed in aerobic reactors treating PAH-contaminated soil (Aburto et al., 2009). The increase of the PAH degradation potential and activity have been related with the presence of the genera *Brevundimonas* and *Pseudomonas* (Chen et al., 2010). Yet, other species of *Brevundimonas* have also been reported as naphthalene oxidizers (El Fantroussi and Agathos, 2005; Rodríguez-Martínez et al., 2006).

High throughput techniques in combination with other approaches, like stable isotope probing (SIP), are very useful for tracing the fate of pollutant compounds. SIP is based on the principle that microorganisms are able to assimilate stable isotope-labeled substrates and incorporate them into the different microbial components (DNA, RNA, proteins, phospholipid fatty acids), which can be further detected by molecular techniques (Neufeld et al., 2007). A long-term coal-tar contaminated groundwater called “Site 24” located in the state of New York, is one of the best characterized fields in terms of PAH contamination and has served as a model for natural attenuation during almost three decades (Madsen et al., 1991; Bakermans et al., 2002; Yagi et al., 2010). Wilhelm et al. (2018) executed a study in “Site 24” to evaluate the natural attenuation process in the affected area. They used a polyphasic approach based on SIP with 13C-napthalene, 16S rRNA gene and shotgun metagenomic sequencing to identify genes and taxa specialized in the naphthalene degradation under oxic (DO < 2.0 mgL^–1^) and hypoxic/suboxic conditions (DO < 0.2 mgL^–1^). The authors observed a naphthalene-degrading bacteria dominance in the microbial community by the decrease in the species richness and Shannon’s diversity index. Due to the increase of about three-fold in the relative abundance of 16S rRNA gene in the metagenomes with 13C-labeled naphtalhene, members of six bacterial genera (*Cupriavidus, Ralstonia, Methylobacterium, Sphingomonas, Stenotrophomonas*, and *Rhodococcus)* were appeared to be responsible for the mineralization and assimilation of 13C-napthalene. However, key genes for the anaerobic conversion of naphthalene by sulfate-reducing bacteria, such as naphthalene carboxylase and naphthoate-CoA, were not present in the metagenomes. On the other hand, genes related in more general anaerobic aromatic degradation pathways were detected, like phenylphosphate carboxylase, benzoate-CoA ligase and succinylbenzoate-CoA ligase. In the search for the genetic basis for alternative redox coupling degradation, the authors found genes related with the dissimilatory nitrate (*narG*) and nitrite (*nirB*) reduction in a metagenome-assembled genome (MAG) affiliated to the *Burkholderiaceae* family. Genes for sulfate and iron reduction were not found in any MAG. Two ring-cleaving dioxygenases (2,3-dihydroxybiphenyl 1,2 dioxygenase and 3-hydrixyanthranilate 3,4-dioxygenase) were present in some MAGs (Wilhelm et al., 2018). The study contributed to gain information about undescribed taxa and metabolic potential associated to the natural attenuation process in the polluted subsurface environment (Wilhelm et al., 2018).

**FIGURE 3:**
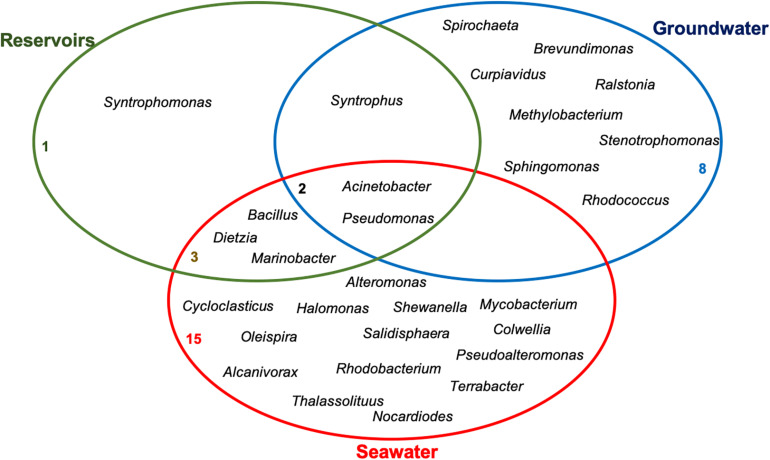
Venn diagram depicting microbial genera that are shared or unique to each environment.

Literature data raised in this review show that there are several genera able to aerobically and/or anaerobically degrade PAHs in the main environments associated to the oil supply chain. A Venn diagram was plotted to show the genera that are exclusive to and those shared between oil reservoirs, seawater and groundwater (Figure 3). Genera like *Acinetobacter* and *Pseudomonas* are common to the three environments reviewed, demonstrating that some of the microorganisms may be considered as generalists, able to grow under different conditions. *Bacillus, Dietzia, and Marinobacter* were reported in seawater samples and oil reservoirs, being the latter specifically found in reservoirs subjected to seawater injection for oil recovery. Genus *Syntrophus* was found in reservoirs and groundwater, showing that syntrophic microorganisms are important to hydrocarbon degradation in these environments. Many exclusive genera like *Cycloclasticus, Halomonas, Colwellia*, among others, were reported in the seawater environment. While *Brevundimonas, Cupriavidus*, *Methylobacterium, Ralstonia, Rhodococcus, Sphingomonas, Stenotrophomonas*, and *Spirochaeta* are exclusive of the groundwater.

## Omics Approaches and PAH Bioremediation

Due to the high environmental contamination caused by the oily supply chain with diverse classes of hydrocarbons, including recalcitrant PAHS, and the urgent need to recover the affected ecosystems, bioremediation has become a topic of intensive research. The main challenges are to make bioremediation processes as efficient as possible and more cost-effective. Considering that the key players in the bioremediation processes are the microorganisms, the great challenge is to improve our knowledge about the biochemical pathways, physiology, ecology, biochemistry and metabolism regulatory mechanisms of the microorganisms responsible for biodegradation, as well as limiting factors affecting petroleum hydrocarbon degradation (Varjani and Upasani, 2017). Anaerobic degradation of PAHs has not yet been well understood so far. However, considering the global extensive use of oil and/or its by-products, and the consequent increase of PAHs polluted environments, it is necessary to pursuit successful clean-up strategies for these contaminants.

Many culturable hydrocarbon degrading bacteria are being isolated based on their capacity to use hydrocarbons as their unique energy and carbon sources, and with genome sequencing it is possible to identify all genes of the degradation pathways. There are many complete or almost complete sequenced genomes of cultured microorganisms which have functional potential metabolism for hydrocarbon degradation (Ghosal et al., 2016). Nevertheless, scientists have long known that only about 1% of the total microbial communities has been cultivated (Hugenholtz and Pace, 1996; Hugenholtz et al., 1998) and advances in the use of -omics techniques, like genomics, proteomics and metabolomics, in bioremediation studies have helped to improve the understanding of biodegradation processes (Brennerova et al., 2009; Nyyssönen et al., 2009; Abbai and Pillay, 2013; Uhlik et al., 2013; Mason et al., 2014; Xu et al., 2014, 2018; El Amrani et al., 2015; Loviso et al., 2015; Ma B. et al., 2015; Techtmann and Hazen, 2016; Zafra et al., 2016; Duarte et al., 2017; Muangchinda et al., 2018; Tiralerdpanich et al., 2018; Wilhelm et al., 2018; Hidalgo et al., 2019). Sequencing metagenomes from diverse contaminated environments (soils, aquifers, seawater) can help to providing insights into the ecology of the dominant and rare members and their functional potential for transformation of pollutant molecules such as PAHs and other hydrocarbons (Blow, 2008; Ghosal et al., 2016). Also, the fast improvements in genome sequencing technology revolutionized the bioremediation application, allowing one to investigate the physiology and ecology of hydrocarbon degrading microorganisms in more detail (Buermans and Den Dunnen, 2014).

Taking into account that environmental conditions can stimulate or inhibit microbial growth, many studies have used microbial community members as bioindicators to detect different kinds of contamination. Based on the microbial community diversity, a model able to quantitatively predict the presence of contaminants in non- polluted and polluted samples was reported (Smith et al., 2015). This predictive model, based on statistical analysis of 16S rRNA biomarker, was initially evaluated in samples from a nuclear waste site and could accurately identify environmental contaminants, as uranium and nitrate. However, the authors extended the model to oil contaminated sites, such as samples from *Deep Horizon* oil spill, in order to explore whether this approach could be applied to other types of pollution. For that, samples collected before and after the oil spill in diverse sampling spots were analyzed. A computational model was developed based on several previously gathered data, allowing to distinguish polluted and unpolluted sites with almost perfect accuracy (98%). They showed that according to the results, the bacteria that showed more accuracy for detecting oil (and uranium) are related with these substrates metabolism, suggesting that the statistical approach was robust and uncover ecological interactions (Smith et al., 2015).

As previously mentioned, the accident involving the *Deepwater Horizon* drilling rig was pioneer oil spill investigated through metagenomics (Crone and Tolstoy, 2010; Reddy et al., 2012; King et al., 2015). Metagenomics was used to improve the comprehension about the fate of oil and the dynamics of biodegradation in the sea water (Techtmann and Hazen, 2016). This spill was the largest one in history and has unique characteristics, due to a remaining oil portion in the deep sediments as a deep water plume of oil (Camilli et al., 2010; Hazen et al., 2010). By using target metagenomics it was possible to investigate the differences in microbial structure and dynamics between deep and surface water (Redmond and Valentine, 2012; Gutierrez et al., 2013). This approach revealed that in the deep-water plume were enrich genes for hydrocarbon degradation and chemotaxis compared to uncontaminated deep water. The use of -omics approaches in the *Deepwater Horizon* oil spill allowed to expand the knowledge of the microbial community reaction to oil spills in the seawater environment as well as the identification of cold-adapted degraders and their role in bioremediation of oil pollution in cold environments (Techtmann and Hazen, 2016). Bioremediation potential, with focus on natural attenuation, is also investigated in marine coastal areas (Catania et al., 2015) and polar soils (Yergeau et al., 2012b). A recent work (Duarte et al., 2017) described a survey about the microbial catabolome for aerobic PAH degradation of a contaminated soil expose to 12 years of *in situ* bioremediation in Czech Republic. The results showed a complex microbial network leading to the discovery of microorganisms similar to those newly identified as *Rugosibacter aromaticivorans* and *Immundisolibacter cernigliae* involved in PAH degradation. Other strategies as bioaugmentation, based on the application of designed microbial consortia, are widely reported in contaminated soils (Zafra et al., 2016; Poi et al., 2018; Haleyur et al., 2019; Wolf et al., 2019) and seawater (Nikolopoulou et al., 2013; Pereira et al., 2019; Shi et al., 2020).

The combination of all these modern molecular biology approaches as metagenomics, metatranscriptomics and metabolomics associated with bioinformatics tools have provided deep insights into the microbial response to PAH pollution, leading to the detection of new metabolic pathways and active degrading microorganisms and contributing to support bioremediation strategies as useful and ecofriendly tools.

## Conclusion

The advances in high throughput sequencing approaches and bioinformatic tools allowed us to improve the understanding of the catabolism of hydrocarbons in several hydrocarbon-rich environments or contaminated sites. These approaches have enabled scientists to characterize and explore microbial communities from different types of affected environments (i.e., groundwater, oil reservoirs, seawater), where the metabolism of PAHs can be differently influenced by various factors (i.e., oxygen concentration, pH, salinity, temperature, nutrient availability, etc.). Although in many studies these strategies have contributed to the identification of key microbial players, genes and mechanisms for PAH degradation, in many other difficult to access environments, the processes remain unidentified. Also, there is currently scarce information about genes and enzymes in anaerobic environments, making the understanding of PAH anaerobic degradation processes, which is prevalent in polluted areas where oxygen is poorly available, even more difficult. Therefore, further innovative and well-designed research is still necessary to completely unveil aspects of PAH biodegradation that remain unknown.

## Author Contributions

KJH, IS-G, and BD: conceptualization, investigation, writing – original draft, and visualization. VO: conceptualization, resources, writing – review and editing, supervision, and funding acquisition. All authors contributed to the article and approved the submitted version.

## Conflict of Interest

The authors declare that the research was conducted in the absence of any commercial or financial relationships that could be construed as a potential conflict of interest.
